# Bayesian Modeling of COVID-19 to Classify the Infection and Death Rates in a Specific Duration: The Case of Algerian Provinces

**DOI:** 10.3390/ijerph19159586

**Published:** 2022-08-04

**Authors:** Hani Amir Aouissi, Ahmed Hamimes, Mostefa Ababsa, Lavinia Bianco, Christian Napoli, Feriel Kheira Kebaili, Andrey E. Krauklis, Hafid Bouzekri, Kuldeep Dhama

**Affiliations:** 1 Scientific and Technical Research Center on Arid Regions (CRSTRA), Biskra 07000, Algeria; 2 Laboratoire de Recherche et d’Etude en Aménagement et Urbanisme (LREAU), Université des Sciences et de la Technologie (USTHB), Algiers 16000, Algeria; 3 Environmental Research Center (CRE), Badji-Mokhtar Annaba University, Annaba 23000, Algeria; 4 Faculty of Medicine, University of Constantine 3, Constantine 25000, Algeria; 5 Department of Public Health and Infectious Diseases, “Sapienza” University of Rome, Piazzale Aldo Moro 5, 00185 Rome, Italy; 6 Department of Medical Surgical Sciences and Translational Medicine, “Sapienza” University of Rome, Via di Grottarossa 1035/1039, 00189 Rome, Italy; 7 Institute for Mechanics of Materials, University of Latvia, Jelgavas Street 3, LV-1004 Riga, Latvia; 8 Department of Forest Management, Higher National School of Forests, Khenchela 40000, Algeria; 9 Division of Pathology, ICAR—Indian Veterinary Research Institute, Izatnagar, Bareilly 243122, India

**Keywords:** Bayesian approach, binomial model, COVID-19, Algeria, mortality and infection rates

## Abstract

COVID-19 causes acute respiratory illness in humans. The direct consequence of the spread of the virus is the need to find appropriate and effective solutions to reduce its spread. Similar to other countries, the pandemic has spread in Algeria, with noticeable variation in mortality and infection rates between regions. We aimed to estimate the proportion of people who died or became infected with SARS-CoV-2 in each provinces using a Bayesian approach. The estimation parameters were determined using a binomial distribution along with an a priori distribution, and the results had a high degree of accuracy. The Bayesian model was applied during the third wave (1 January–15 August 2021), in all Algerian’s provinces. For spatial analysis of duration, geographical maps were used. Our findings show that Tissemsilt, Ain Defla, Illizi, El Taref, and Ghardaia (Mean = 0.001) are the least affected provinces in terms of COVID-19 mortality. The results also indicate that Tizi Ouzou (Mean = 0.0694), Boumerdes (Mean = 0.0520), Annaba (Mean = 0.0483), Tipaza (Mean = 0.0524), and Tebessa (Mean = 0.0264) are more susceptible to infection, as they were ranked in terms of the level of corona infections among the 48 provinces of the country. Their susceptibility seems mainly due to the population density in these provinces. Additionally, it was observed that northeast Algeria, where the population is concentrated, has the highest infection rate. Factors affecting mortality due to COVID-19 do not necessarily depend on the spread of the pandemic. The proposed Bayesian model resulted in being useful for monitoring the pandemic to estimate and compare the risks between provinces. This statistical inference can provide a reasonable basis for describing future pandemics in other world geographical areas.

## 1. Introduction

The new coronavirus, severe acute respiratory syndrome coronavirus 2 (SARS-CoV-2), which can cause acute respiratory disease in humans, emerged in late 2019 as a new global epidemic [1]. The virus originated in Wuhan, a city in China’s Hubei province, and was identified in late December 2019 as responsible of the coronavirus 2019 disease (COVID-19) [2]. Currently, more than 576 million confirmed cases and over 6.4 million deaths have been reported due to COVID-19 as of 30 July 2022 [2]. The entire world population is currently facing great challenges (particularly socio-economic). As an example, in Italy this emergency led to a structural and organic deficiency that impacted on both the costs of managing infections in healthcare facilities and peoples’ health related behaviors [3,4]. A study from USA showed that recent COVI-19 related job loss causes were significantly related with suicide specifically in the lockdown phase [5]. In Spain, it caused a declining economic activity while expenditure must rise to combat the infection and its social and economic consequences generated huge public deficits hard to finance [6]. According to Elkhashen et al. [7], the pandemic’s primary impact on the Egyptian economy was the slowdown in all domestic activities and the significant fall in income from tourism, remittances, and Suez Canal have severely eroded household incomes, pushing millions of people in poverty.

Moreover, the emerging SARS-CoV-2 variants have resulted in multiple waves of pandemic over the time. At the moment, a vast surge in COVID-19 cases is being observed worldwide, mainly due to the recently emerged Omicron (B.1.1.529) variant of concern [2,8].

Therefore, studying the pandemic is essential for acquiring comprehensive analytical knowledge about the new phenomenon and finding appropriate measures to control the spread of the disease [9,10], also based on previous experience in infectious diseases control [11]. Global efforts have resulted in identifying several antiviral drugs, and a few vaccines have been developed [12,13], however, no clinically approved treatments have been identified, despite many trials of drug repurposing [14,15].

Each affected country reacted in managing the spread of the disease mainly through self-distancing and lockdown policies, increasing testing, vaccination, and treatment, and reducing large-scale meetings [16].

In recent years, the application of mathematical models in epidemiology has increased [17], showing the importance of interdisciplinary. Mathematical and health science employ models as tools to analyze data and direct decision-making. Mathematical model construction makes it easier to conduct thorough analysis and enables quantitative forecasting of changes in disease burden and the effects of interventions [17].

With regard to Africa, several studies have been conducted on COVID-19 spread and modelling. Ababsa et al. examined the spread of COVID-19 and the effect of climatic factors in Algeria [18]. Fatih et al. investigated the transmission of the virus in Algeria, Egypt, and South Africa [19]. Kadi et al. studied the association between population density and the spread of COVID-19 in Algerian cities [20]. Other authors used the susceptible-infected-recovered (SIR) model to predict the daily number of COVID-19 cases in Algeria [21].

However, no previous studies shed light on the arrangements of the countries by the infection rate or death rate due to the virus.

With the rapid development of contemporary science, the Bayesian paradigm becomes an asset, since it offers reasoning well suited to the use of the different sources of information involved in decision-making in an environment of uncertainty. The Bayesian approach appears as a powerful and solidly integrated approach in the new technology, where the relations exist between information technology [22]. As a matter of fact, the Bayesian paradigm makes it possible to integrate a priori information in a natural way, unlike the frequentist paradigm [23].

Bayesian modeling also captures a priori information to rank provinces of a country based on the epidemic spread and death rates [18,19,20,24].

Estimation of the Bayesian model using OpenBUGS (Supplementary Materials) is made with the greatest number of iterations to produce an estimate that is more accurate and closer to the true values of the parameters by providing OpenBUGS software codes through which other users can analyze, with a priori information, and subsequently develop these packages.

To our knowledge, no studies were conducted on COVID-19 using the Bayesian approach in Algeria, though there are some examples of using this approach from Africa such as in Morocco [25], Tunisia [26], and Egypt [27].

This study aims to identify the spatial distribution of COVID-19 in Algeria, using the Bayesian approach. This spatial analysis classifies provinces according to the risk of contagion and death, which makes it possible to understand the relationship between different regions during different epidemic stages and enables national authorities to contribute to the fight against this pandemic in a most effective and efficient way. This study also aims to build a stable predictive model to predict the probability of infection and severity that can be adapted to other countries.

## 2. Materials and Methods

A model for estimating epidemic risks and measuring their magnitude between the different Algerian provinces during a given period was developed.

### 2.1. Study Period

This study covers the third wave of the COVID-19 pandemic (between 1 January 2021, and 15 August 2021) and classifies provinces according to the risk of infection and death.

### 2.2. Data Acquisition

The data were obtained, on request, from the Algerian Ministry of Health. The data included a 227-days-period (from 1 January 2021 to 15 August 2021), the number of confirmed cases, and the number of deaths. In addition, these data were divided into 48 provinces, and the total number of infections and deaths for each day during this period was calculated.

**Figure 1:**
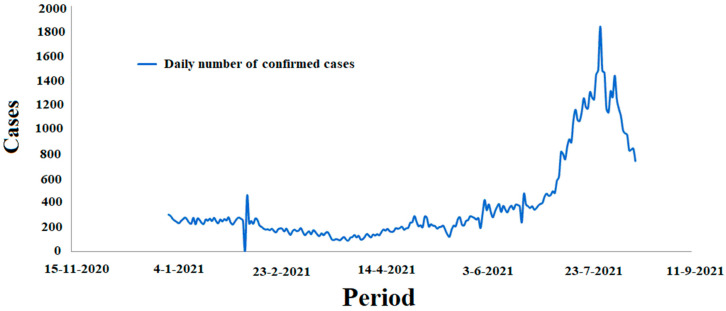
The number of COVD-19 cases in Algeria in the period between 1 January 2021 and 15 August 2021.

**Figure 2:**
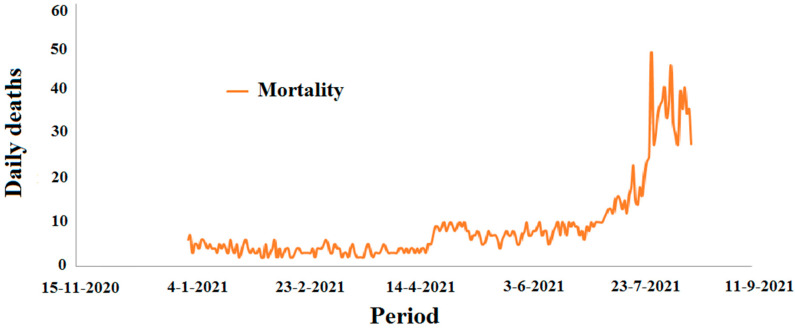
Mortality due to COVD-19 in Algeria in the period between 1 January 2021 and 15 August 2021.

Figure 1 and Figure 2 report the incidence of confirmed cases and the mortality rate by COVID-19 in Algeria, respectively.

### 2.3. Statistical Analysis

#### 2.3.1. Bayes’ Formula and Posterior Distribution

The Bayesian concept differs from the classical ones because the parameter has become a random variable with “a priori distribution” [28,29,30]; through this conception, the analysis allows to consider all the qualitative and quantitative information on the uncertainty in the model. Then, using Bayes’ theorem, which allows making parameters as values of random nature, it is possible to deduce the distribution “a posteriori”, which allows us to construct inferential procedures in the most natural way. In the Bayesian approach, all priors are informative in some way [28,29,30].

If we consider *n* disjoint hypotheses (*H*) being mutually exclusive and temporarily exhaustive, at any point in time, it is possible to collect all hypotheses that have been constructed, in light of available knowledge, into an hypothesis set $H_{i}$ ($H_{1},H_{2},\ldots ,H_{n}$) [28,29,30]. As more information is gathered, the competing hypotheses would progressively surface. The “correct” hypothesis is difficult to get, but there is a tendency to accept one of the alternatives, as being more likely than the others. In other words, compared to the alternatives, there may be a greater degree of certainty/uncertainty that one hypothesis is correct. The uncertainty is described in terms of probability in Bayesian analysis. In this situation, the occurrence of an event E of non-zero probability is represented as:

(1) $$E=\left(E⋂H_{1}\right)⋃\left(E⋂H_{2}\right)⋃\ldots ⋃\left(E⋂H_{n}\right)\;$$

According to the theory of total probabilities [31], the probability of the event *E* is:

(2) $$P\left(E\right)=P\left(E⋂H_{1}\right)+P\left(E⋂H_{2}\right)+\cdots +P\left(E⋂H_{n}\right)\;$$

If we consider $P\left(H_{i}\right)$ as the probability assigned to the “hypothesis $H_{i}$”, and $P\left(E/H_{i}\right)$ the conditional probability [13] of the event observed under hypothesis $H_{i}$, it is possible to consider the probability of observing the event P(E), over all possible hypotheses, as:

(3) $$P\left(E\right)=\overset{n}{\underset{i=1}{\sum }}P\left(E/H_{i}\right)P\left(H_{i}\right)=E^{H}\left(P\left(E/H_{i}\right)\right)$$

where $E^{H}$ indicates an expectation taken with respect to the prior distribution of the hypotheses.

Using the last two formulas, it is possible to get the last definition for $P\left(E/H_{i}\right)$:

(4) $$P\left(H_{i}/E\right)=\frac{P\left(E⋂H_{i}\right)}{P\left(E\right)}=\frac{P\left(E/H_{i}\right)P\left(H_{i}\right)}{\sum_{i=1}^{n}P\left(E/H_{i}\right)P\left(H_{i}\right)}\;$$

The validity of the Formulas (1)–(4) have already been demonstrated and is not reported in depth [32].

This standard conditional probability conclusion may refer to the *Bayes theorem*, or “inverse probability”. According to this, the likelihood of a hypothesis $H_{i}$, given event E, is inversely correlated with the product of the prior probability assigned to the hypothesis, $P(H_{i})$ and the conditional probability of witnessing the event E under hypothesis $H_{i}$.

Bayes used the continuous version of this theorem, taking two random variables x and y. Therefore, the conditional distribution of y knowing x is given by:

(5) $$\pi \left(y/x\right)=\frac{f\left(x/y\right)\times f\left(y\right)}{\int^{\;}f\left(x/y\right)\times f\left(y\right)dy}\;$$

Equation (5) allows us to make inferences from the distribution of the parameter θ conditional on the observations x, called the a posteriori distribution [33], and is defined by:

(6) $$\pi \left(\theta /x\right)=\frac{f\left(x/\theta \right)\times \pi \left(\theta \right)}{\int_{\theta }f\left(x/\theta \right)\times \pi \left(\theta \right)d\theta }=\frac{f\left(x/\theta \right)\times \pi \left(\theta \right)}{m\left(x\right)}\;$$

We pose the marginal distribution of x:

(7) $$m\left(x\right)=\underset{\Theta }{\int }f\left(x/\theta \right)\pi \left(\theta \right)d\theta \;$$

This a posteriori distribution is the combination of:

f(x/θ): the density function of x knowing the value of the random variable θ;
π(θ): the a priori density function on θ;
m(x): the marginal distribution of x.

Equation (6) represents what is known and unknown before, with respect to the parameter considering the observed data [34]. Moreover, it is an update of π(θ) after the observation of our sample.

Once the data are available, the amount of m(x) is a normalization constant that guarantees that π(θ/x) is a posteriori probability distribution. Therefore, π(θ/x) is directly proportional to f(θ/x)×π(θ), which means that the Bayesian inference verifies the likelihood principle “a posteriori”, and the information from the data comes exclusively from the likelihood f(x/θ).

It is frequent to construct a logarithm to simulate the correlated observations, so one relies on the internal process of the simulated samples. For this objective, we used the Markov chain Monte Carlo method [35,36] and the natural conjugate laws [37].

#### 2.3.2. The Estimation Model

It is assumed that the number of deaths or infections in the considered time interval is a realization of a binomial distribution, written respectively as:

(8) $$dc_{i}\sim \beta in\left(n_{1i},q_{1i}\right)$$

(9) $$Ic_{i}\sim \beta in\left(n_{2i},q_{2i}\right)$$

where, $n_{1i},n_{2i}$ are the total number of deaths and infections, respectively, in the study period for province i, and $q_{1i},q_{2i}$ are the probability (proportion) of deaths or infections, respectively, during the study period for province i.

The application of Bayes’ theorem appears to be an “updating” principle. From the likelihood function of a sample of *m* individuals, it is possible to obtain the posterior probability (proportion) of deaths or infections, respectively, during the study period for province i:

(10) $$\pi \left(q_{1i}/dc_{i}\right)\;\mathrm{is}\;\mathrm{directly}\;\mathrm{proportional}\;\mathrm{to}\;f\left(dc_{i}/q_{1i}\right)\pi \left(q_{1i}\right)$$

(11) $$\pi \left(q_{2i}/Ic_{i}\right)\;\mathrm{is}\;\mathrm{directly}\;\mathrm{proportional}\;\mathrm{to}\;f\left(Ic_{i}/q_{2i}\right)\pi \left(q_{2i}\right)$$

The probabilities of death (from infection) are considered independent, and identically distributed (i.i.d). From a Bayesian point of view, we assume an a priori distribution for $q_{i}$. As the natural conjugate of a binomial distribution is a beta prior, we obtain:

(12) $$q_{i}\sim ℬℯ\left(\alpha ,\beta \right)\;$$

The flexibility of the form of the beta distribution, the ease of constructing the a posteriori distribution, and the support of the distribution allow an analysis of the different phenomena. In the case of a uniform measure with respect to the Lebesgue measure [38], the a priori distribution is not invariant by re-parametrization. Generally, we obtain:

(13) $$q_{i}\sim ℬℯ\left(1,1\right)\;$$

A conjugate distribution can be determined by considering the form of the likelihood f(x⁄θ) and by taking a prior distribution of the same form as the latter. The conjugate prior laws obtained by this process are known to be the natural conjugate laws [38].

In this model, we assume different locations (i.e., a difference between the provinces), the same for the whole duration between the days i=1,…m, and for a province denoted by j such that j=1,…,48, we can write, respectively:

(14) $$dc_{ij}\sim \beta in\left(n_{1j},q_{1j}\right)$$

(15) $$Ic_{ij}\sim \beta in\left(n_{2j},q_{2j}\right)$$

and:

(16) $$q_{1j}\sim ℬℯ\left(1,1\right)$$

(17) $$q_{2j}\sim ℬℯ\left(1,1\right)$$

## 3. Results

### 3.1. Modeling Mortality Rates

The death rate due to the virus in each province was calculated by dividing the number of deaths due to the virus in one province during all the studied period and dividing it by the number of deaths in the whole country. The COVID-19 mortality rates in the counties are shown in Table 1. To approach this, we employed OpenBUGS with a number of 30,000 iterations in order to perform MCMC simulations to calculate Equation (8), considering the great quantity of data to be analyzed.

**Figure 3:**
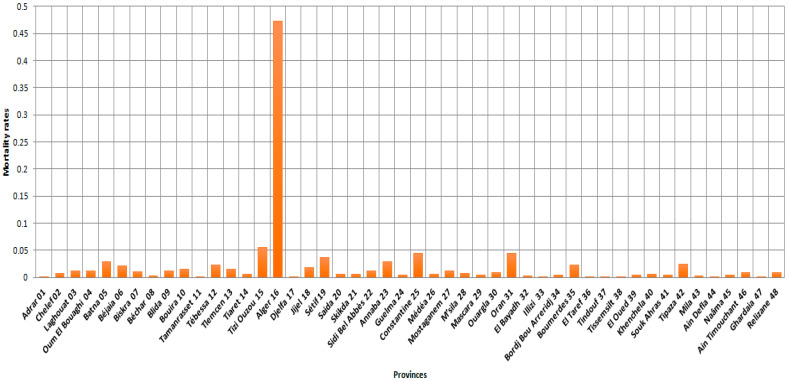
Provincial mortality rates (48 provinces) by COVID-19 between 1 January 2021 and 15 August 2021.

According to Figure 3, Algiers province had the highest mortality rate, which means that the risk of death in this province by COVID-19 between 1 January 2021, and 15 August 2021, is nine times higher than the other provinces (Tizi Ouzou and Oran).

**Figure 4:**
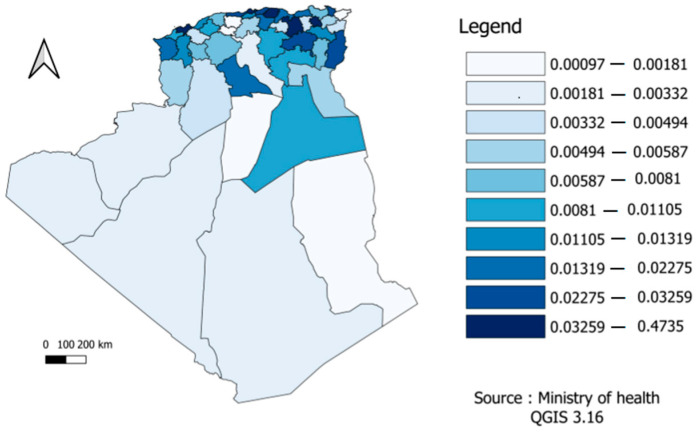
Distribution of COVID-19 mortality risk by provinces.

In the figure below, the mortality risk was divided according to the different provinces into ten sections or levels in order to facilitate reading and clarify the content. According to Figure 4, we found differences across the Algerian regions. For example, the northeast, where most of the population is concentrated, was the region that suffered the most deaths.

**Figure 5:**
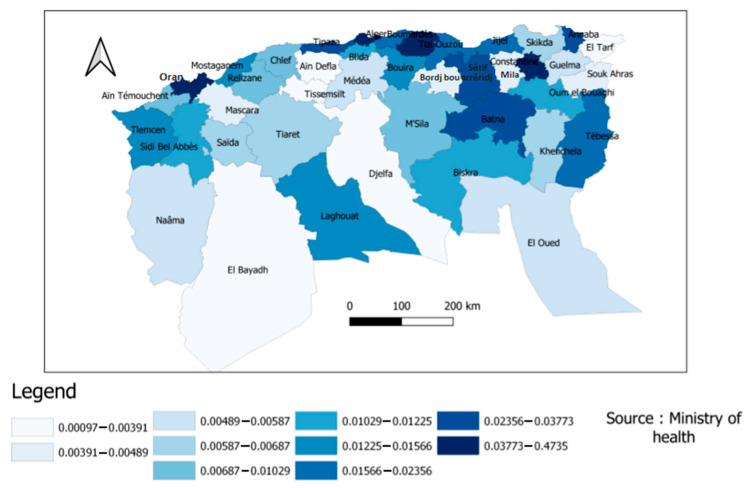
Distribution of COVID-19 mortality risks in the Northern provinces.

According to Figure 5, most provinces with high COVID-19 mortality rates were the most populated.

Thanks to the advent of marginal distributions in the use of the Bayesian approach, the classification of mortality risks between provinces was possible using several statistic measures (the mode, the median, etc.). In our analysis, we used the a posteriori mode in the classification, which gave decimal digits (without commas).

**Figure 6:**
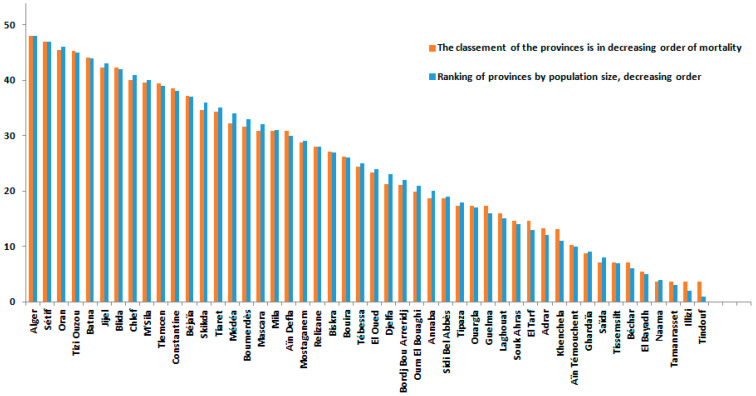
Classification of mortality rates in Algerian provinces (48 provinces) by COVID-19 between 1 January 2021 and 15 August 2021.

**Figure 7:**
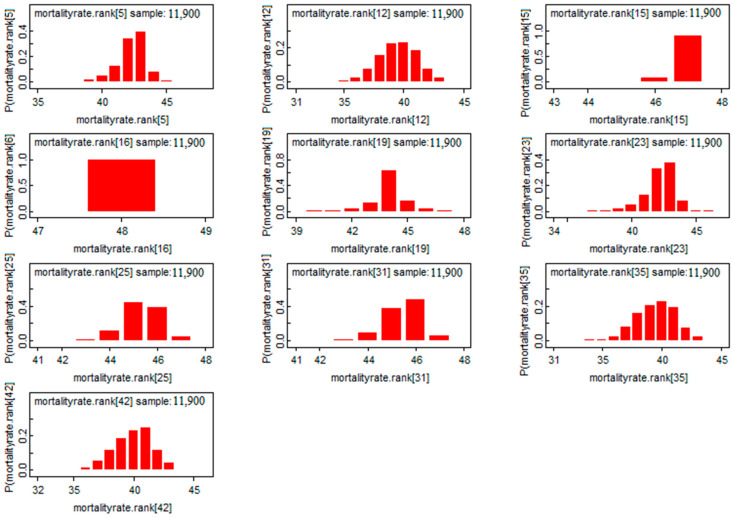
Risk classification of the mortality rate of the top 10 provinces.

Figure 3, Figure 6 and Figure 7 provided a more accurate and detailed view of the level of mortality risks by province. The provinces of Batna and Tizi Ouzou showed higher risks, and this means that besides the population density component already demonstrated [20], there are other factors related to the distribution of the number of deaths in the country [10]. The three provinces with the largest population density (Algerirs, Oran, and Constantine) were at the forefront of the ranking with the provinces of Tizi Ouzou, Setif, and Batna.

**Figure 8:**
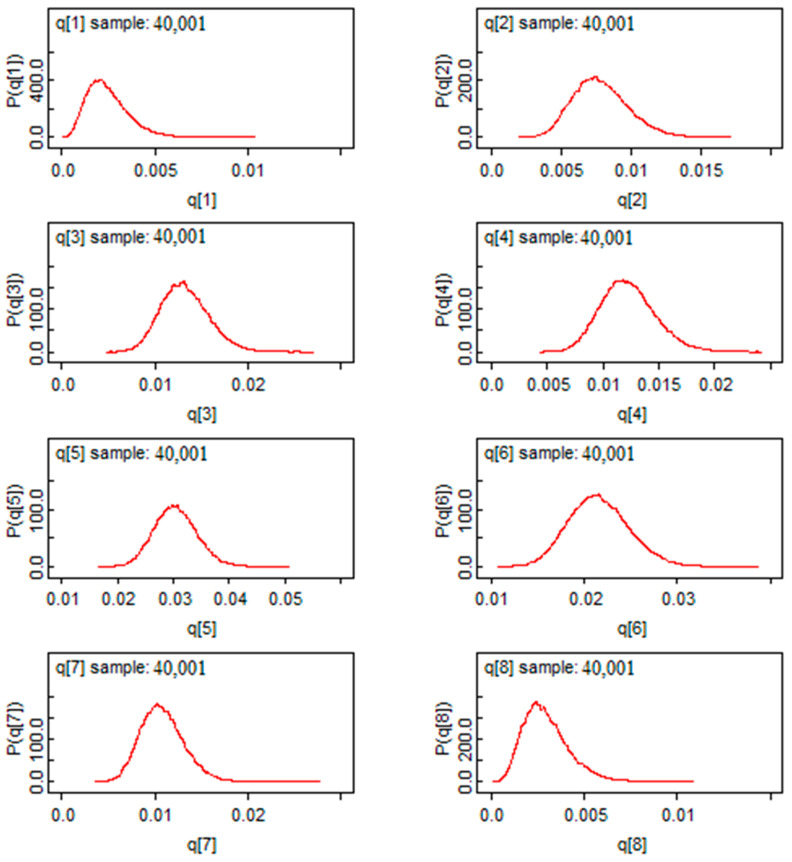
The post hoc risk distribution for the first 8 provinces.

According to Figure 8, we found a Gaussian shape for the mortality rate estimators for the first eight provinces. This exhibits a good sign of convergence for this model. Moreover, marginalization allows Bayesian analysis to eliminate nuisance parameters, in another way, it reduces the dimension of the parameter “space” to be estimated. Based on this characteristic, we found marginal distributions in Figure 8. Through these distributions, the posterior mass is provided and the comparison between the risks will be easier. In Figure 8, we saw that all the a posteriori values are located to the right of the value “0”, which means that the risk of mortality is significant in the provinces represented.

### 3.2. Modeling of Infection Rates

The rate of infections due to the virus in each province was calculated by dividing the number of cases infected with the virus in one province during all the study period and dividing it by the number of infections in the whole country.

**Figure 9:**
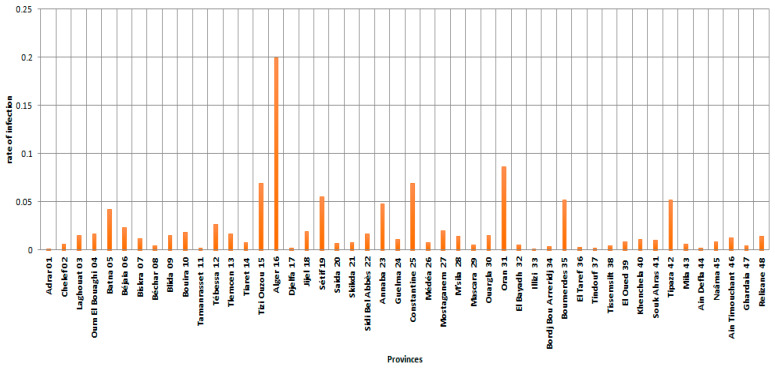
Infection rates in the provinces (48 provinces) by COVID-19 between 1 January 2021 and 15 August 2021.

**Figure 10:**
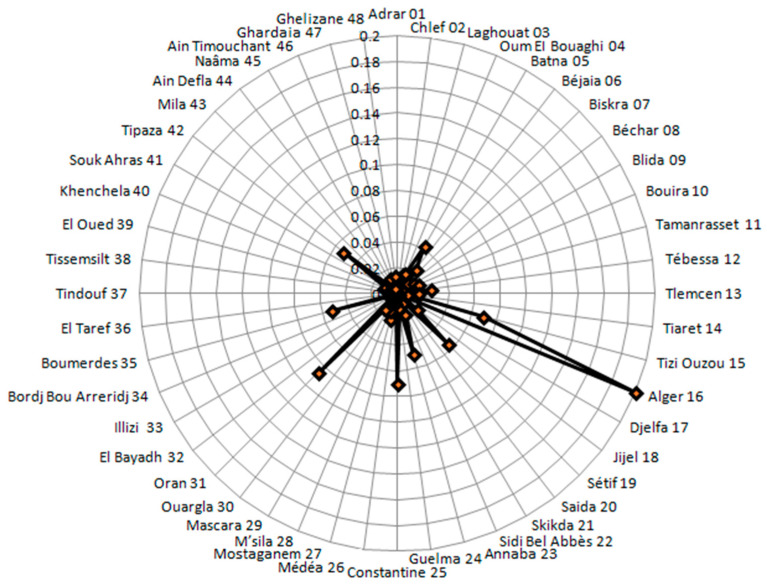
Radar plot of Morbidity rates in the provinces (48 provinces) by COVID-19 between 1 January 2021 and 15 August 2021.

Figure 9 and Figure 10 showed the rates of infection in different provinces. In particular, the radar chart in Figure 10 provides an illustrative data for the spread of the virus within the scope of the country, or even a group of countries in some cases. It is evident that the majority of the provinces were concentrated within a band, which means that the dispersion is low in most of the provinces with regard to infection, which is more prominent in the provinces of Algiers, Annaba, Tizi Ouzou, Setif, Constantine, Oran, Tipaza, Boumerdes, and Batna. Figure 9 and Figure 10 also showed that Algiers Province had a very high peak infection rate, indicating that the risk of COVID-19 infection in this province between 1 January 2021 and 15 August 2021 is higher than the risk in the other provinces (Tizi Ouzou, Sétif, Constantine, and Oran provinces).

Table 2 presented the COVID-19 infection rates in the country, where we used OpenBUGS with 30,000 iterations in order to use Equation (8) on a large amount of data.

Table 2 showed that the provinces of the Sahara are the least affected by COVID-19. Moreover, the risk of infection in Algiers province was twice higher than in Oran province. This observation, together with the mortality rate, indicated a mutual risk of mortality and infection in this province.

**Figure 11:**
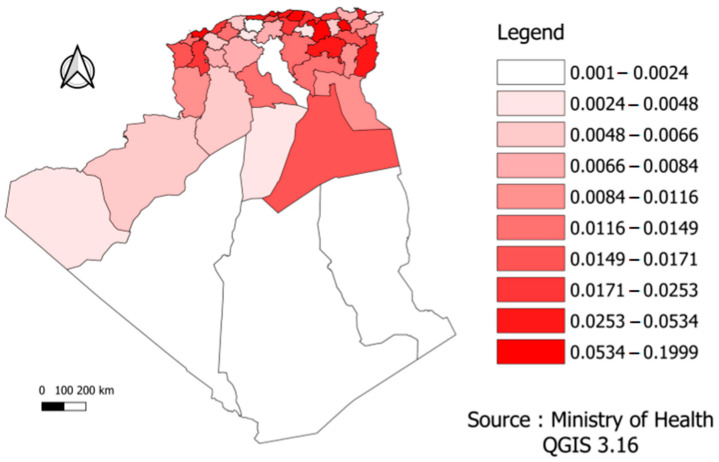
Risk distribution of COVID-19 infection by province.

In the above figure, the infection risk was divided according to the different provinces into ten sections or levels in order to facilitate reading and clarify the content. Figure 11 showed the distinction across the Algerian regions. The northeast, where the population is concentrated, was the most affected region by COVID-19 and had the highest infection rate. The province of Ouargla in southern Algeria also showed a high infection rate, which may result from economic activity (specifically related to oil and gas, as they are the main products of this province). The economy may be the most important explanatory factor of this finding.

**Figure 12:**
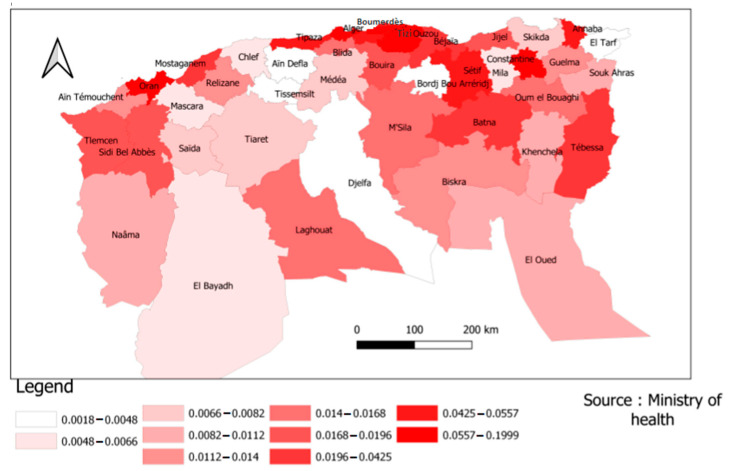
Risk distribution of COVID-19 infection in the Northern provinces.

According to Figure 12, the province of Laghouat presented a significant risk of infection, whereas the province of Djelfa does not. This means that the infection rate was distributed in a random manner between Algeria’s northern and central provinces.

**Figure 13:**
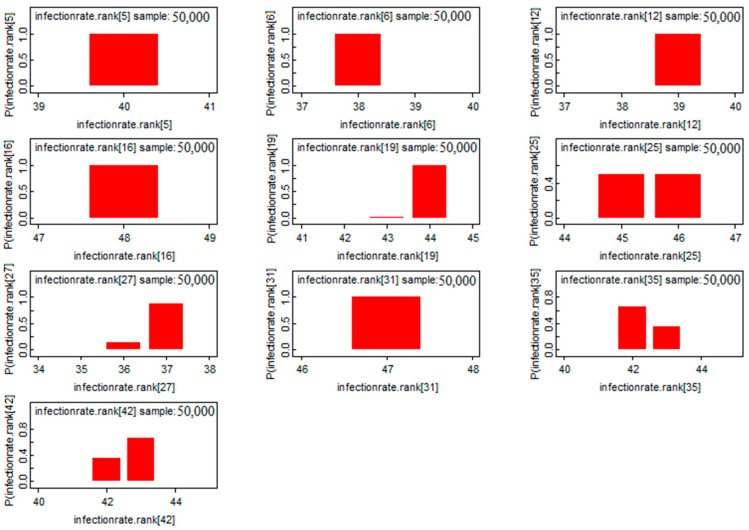
Risk classification of the COVID-19 infection rate of the top 10 provinces.

**Figure 14:**
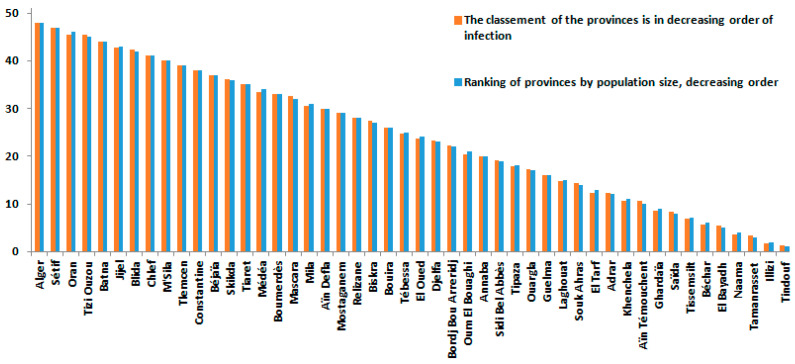
Classification of infection rates in Algerian provinces (48 provinces) by COVID-19 between 1 January 2021 and 15 August 2021.

Figure 13 and Figure 14 provided a more accurate view of the risk of infection by province. The provinces of Tizi-Ouzou, Boumerdes, Annaba, Tipaza, and Tébessa showed higher risks in relation to their population size, which means that the rate of infection between provinces is based on several explanatory variables beyond the size of the population, economic activity, and even distance from the borders.

The classification of infection risks between regions using a variety of statistics is now straightforward due to the development of marginal distributions in the application of the Bayesian technique (the mode, the median, etc.). In our investigation, we classified data using the mode method given the shape of the marginal distributions.

**Figure 15:**
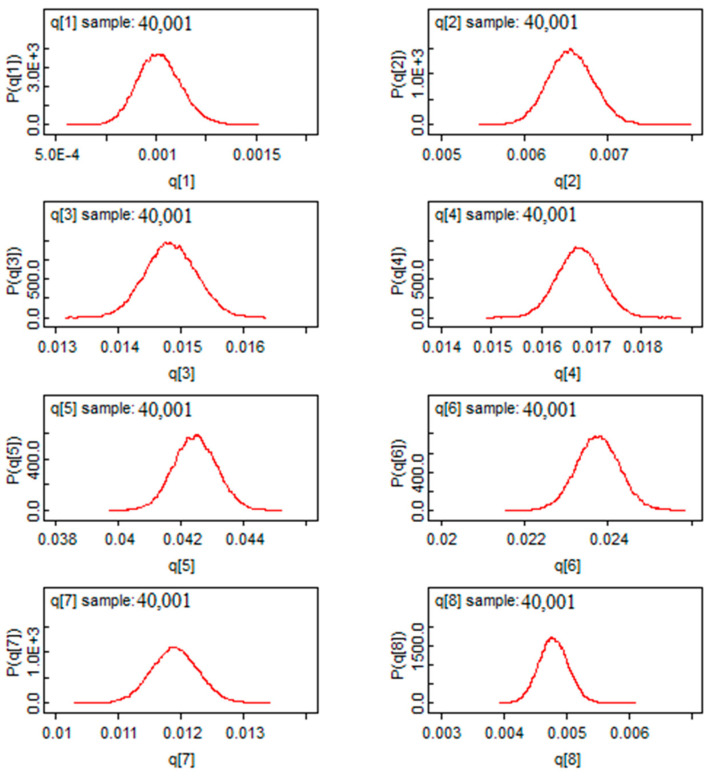
The post hoc distribution of infection rates for the first provinces.

In Figure 15, the infection rates showed a Gaussian shape of the estimators, showing a good sign of convergence for the Bayesian estimators by using the Bayes factor. This method allows us to conduct parametric tests of comparison between the risk of infection and death in two separate provinces.

## 4. Discussion

This study presents COVID-19 related infection and death models in Algeria between 1 January 2021 and 15 August 2021, with the aim of investigating the differences between provinces. To our knowledge, this is the first study in Algeria to aggregate a large amount of data into clear and readable metrics over a seven-month period, whereas past studies are limited to a single day analysis [39].

It is well known that the SIR model and its variants have been commonly applied to the current COVID-19 outbreak. Tang et al. [24] investigated an estimated epidemiological model for COVID-19 based on a classic susceptible-exposed-infected-recovered (SEIR) model. Wu et al. [40] proposed an extended SEIR model to predict the spread of COVID-19 within and outside Mainland China. However, both models suggested that the exposed population was not contagious, which may not be sufficient for COVID-19 spreading. Yang et al. [41] predicted the epidemic trend of the virus outbreak in China using a combination of the SEIR model and a machine-learning artificial intelligence approach.

In general, the strength of a model is not measured by its complexity, degree of statistical significance, or statistics of model choice, and there is usually no statistical model that is entirely correct; however, they offer useful research tools [42,43]. In this study, we have considered the nature of biased data in Algeria, which may be neglected in previous studies [44]. By using the proportional relationships between the number of infections and the number of deaths at other times, the result was the genesis of a consistent model with reality. This can be very useful in decision making, since it is currently undeniable that managing pandemics is always difficult [45,46].

In our study, we observed that the provinces of Tissemsilt, Ain Defla, Illizi, El Taref, and Ghardaia were the least affected by COVID-19 mortality. These findings are different from the results of Kadi and Khalfaoui [20], showing that the cities that were, until now, considered the most spared are characterized by high mortality in the cluster analysis of the first wave. In addition, the risk of infection in Algiers province is twice as high as that in Oran province (the second largest city of Algeria), which is consistent with previous studies [47]. Our results showed that the risk of death in the province of Algiers from COVID-19 during the study period was nine times greater than that in the other provinces, which reflects the reality given that Algiers is the largest and most populous city in Algeria and a centralized place of ministries and important institutions [48].

In contrast, the province of Tizi Ouzou has a high risk of death from COVID-19, despite its medium population density. To date, no study has reported this in Algeria, and the reasons can be multiple. Nevertheless, some studies (mainly from Canada and the USA) showed that suburban areas had higher rates than the major larger city [49,50,51]. In our case, the most logical explanation is non-compliance with the protective measures, especially in a political and social context historically tense in this geographical area [33]. Another expected result comes from the fact that in the Algerian regions in the northeast, where the population is concentrated, is the region with the highest rate of COVID-19 infection. This finding is confirmed by studies focusing on larger geographical areas in Algeria [52]. Nonetheless, there are many other factors that could explain a medium size population experiencing higher rates of COVID-19, such as movements of population (e.g., immigrants and tourists). The province of Ouargla in southern Algeria shows a high infection rate, which may be the result of economic activity in this province and the influx of illegal immigration [53], underlining the importance of screening for infectious diseases [54]. It is worth noting that according to this study, population size is not a linear factor. Other factors may influence the spread of the epidemic and its relative mortality, such as economic activity and geographical location [55]. Indeed, the spatial maps in this study are not an exhaustive solution, but they can provide information on the means to study infection and mortality rates in a simple form and via the Bayesian approach, where a priori information can be incorporated, if available. In the present study, spatial point analysis provides concentrated information and makes us better understand COVID-19 waves and their propagation, which has never been tackled in Algeria. According to Figure 10, the provinces of Algeria, Sétif, Annaba, Tipaza, Oran, Constantine, Boumerdes, Bejaia, and Tizi Ouzou represent regions where the epidemic is most widespread, which is closer to the reality and is in line with previous findings [56]. The Bayesian Markov chain Monte Carlo (MCMC) method confirms that it is relatively easy to implement and provide a set of suitable techniques for estimating duration models [57].

The results must be interpreted within the limitations of the methods used. Undoubtedly, this study introduces an analysis model based on Bayesian theory that needs to be further tested and demonstrate its validity. Moreover, we found that the point of vulnerability lies in the absence of explanatory (independent) variables. It is important to note that the findings may be biased by variables not included in the study. Based on our findings, further studies are needed, using as many explanatory variables as possible within a multivariate model. In addition, the quality of the model prediction depends on the availability of data. In our case, as specified in the methodology section, the data acquired to implement this study were from official sources (Algerian Ministry of Health). Given that the number of infections was based on PCRs only, the official number of cases is surely underreported.

This study can be the basis for improving the use of modeling in general, and Bayesian methods in particular, for researchers who work on COVID-19, not only in Algeria.

## 5. Conclusions

This study provides several important insights. Factors affecting mortality due to COVID-19 seem not necessarily dependent on the spread of the pandemic. This remark is evident when comparing the rankings of the provinces by the risk of death or infection. The two rankings are partially equivalent. Moreover, the used methodology contributes to scientific research on Bayesian analysis concerning the risk assessment of infection and mortality applied to the current COVID-19 pandemic. In addition, the proposed Bayesian model is a useful tool for both monitoring the pandemic and estimating and comparing the risks across provinces of a country. Updating the estimates daily would make the tool more efficient and useful. Finally, this type of statistical inference can be a reasonable basis for possible use to describe future pandemics in other geographical areas, given that the proposed model can be easily implemented in other countries and regions.

## Figures and Tables

**Table 1:** Mortality rate due to COVID-19 in the provinces ^1^.

Provinces	Mean	Provinces	Mean	Provinces	Mean	Provinces	Mean
Adrar	0.002	Tlemcen	0.016	**Constantine**	**0.045**	Tindouf	0.002
Chelef	0.008	Tiaret	0.007	Médéa	0.006	Tissemsilt	0.001
Laghouat 0	0.013	**Tizi Ouzou**	**0.056**	Mostaganem	0.013	El Oued 39	0.005
Oum El Bouaghi	0.012	**Alger**	**0.474**	M’sila	0.008	Khenchela	0.006
Batna	0.03	Djelfa	0.002	Mascara 9	0.004	Souk Ahras	0.005
Béjaia	0.022	Jijel	0.019	Ouargla	0.009	Tipaza	0.025
Biskra	0.011	Sétif	0.038	**Oran**	**0.046**	Mila	0.004
Béchar	0.003	Saida	0.006	El Bayadh	0.004	Ain Defla	0.001
Blida	0.012	Skikda	0.007	Illizi	0.001	Naâma	0.005
Bouira	0.015	Sidi Bel Abbès	0.012	Bordj Bou Arreridj	0.004	Ain Timouchant	0.01
Tamanrasset	0.002	Annaba	0.03	Boumerdes	0.024	Ghardaia	0.001
Tébessa 2	0.024	Guelma	0.005	El Taref	0.001	Relizane	0.01

^1^ The most affected provinces are indicated in bold black.

**Table 2:** Provincial COVID-19 infection rates ^1^.

Provinces	Mean	Provinces	Mean	Provinces	Mean	Provinces	Mean
Adrar	0.0010	Tlemcen	0.0170	**Constantine**	**0.0694**	Tindouf	0.0024
Chelef	0.0066	Tiaret	0.0078	Médéa	0.0076	Tissemsilt	0.0048
Laghouat	0.0148	**Tizi Ouzou**	**0.0694**	Mostaganem	0.0204	El Oued	0.0086
Oum El Bouaghi	0.0168	**Alger**	**0.1999**	M’sila	0.0143	Khenchela	0.0112
Batna	0.0425	Djelfa	0.0018	Mascara	0.0053	Souk Ahras	0.0107
Béjaia	0.0238	Jijel	0.0196	Ouargla	0.0152	Tipaza	0.0524
Biskra	0.0119	Sétif	0.0557	**Oran**	**0.0861**	Mila	0.0064
Béchar	0.0048	Saida	0.0071	El Bayadh	0.0053	Ain Defla	0.0024
Blida	0.0155	Skikda	0.0082	Illizi	0.0011	Naâma	0.0084
Bouira	0.0186	Sidi Bel Abbès	0.0171	Bordj Bou Arreridj	0.0041	Ain Timouchant	0.0129
Tamanrasset	0.0019	Annaba	0.0483	Boumerdes	0.0520	Ghardaia	0.0041
Tébessa	0.0264	Guelma	0.0113	El Taref	0.0030	Relizane	0.0141

^1^ The four most affected provinces are indicated in bold black.

## Data Availability

The data presented in this study are available on request from the corresponding author.

## Supplementary Materials

The following supporting information can be downloaded at: https://www.mdpi.com/article/10.3390/ijerph19159586/s1, OpenBUGS Project Definition and The OpenBUGS codes.
