# Supplementary material for: Bayesian Modeling of COVID-19 to Classify the Infection and Death Rates in a Specific Duration: The Case of Algerian Provinces
Source: Int J Environ Res Public Health. 2022 Aug 4;19(15):9586. doi: 10.3390/ijerph19159586 (PMC9368112; doi:10.3390/ijerph19159586)
Supplement: Supplementary file 1 [file ijerph-19-09586-s001.zip › ijerph-1766303-supplementary.pdf]

International Journal of
Environmental Research and Public Health

MDPI

Supplementary Materials

S1. BUGS Project Definition

BUGS is a software package for performing Bayesian inference using Gibbs Sampling. The project started in Cambridge in 1989. BUGS made Bayesian analysis accessible to anyone using a laptop computer. Bayesian analysis could only be applied in the pre-BUGS era in situations where solutions could be obtained in closed form in so-called conjugate analyses, or by an ingenious but limited application of numerical integration methods. As a result, BUGS has helped raise awareness of Bayesian modeling in the academic and commercial communities.

S2. The OpenBUGS codes

OpenBUGS represents the free and open extension of WinBUGS.

The code for OpenBUGS or WinBUGS software used for estimating infection rates (the same procedure used in estimating death rates) is given in this appendix.

The beta-Binomial model for estimating infection rates

```
model
{
for (i in 1:m) {
d[i,1]~dbin(q[1],n[i])
}
q[1]~dbeta(1, 1)
for (i in 1:m) {
d[i,2]~dbin(q[2],n[i])
}
q[2]~dbeta(1, 1)
for (i in 1:m) {
d[i,3]~dbin(q[3],n[i])
}
q[3]~dbeta(1, 1)
for (i in 1:m) {
d[i,4]~dbin(q[4],n[i])
}
q[4]~dbeta(1, 1)
for (i in 1:m) {
d[i,5]~dbin(q[5],n[i])
}
q[5]~dbeta(1, 1)
for (i in 1:m) {
d[i,6]~dbin(q[6],n[i])
}
q[6]~dbeta(1, 1)
for (i in 1:m) {
d[i,7]~dbin(q[7],n[i])
}
q[7]~dbeta(1, 1)
for (i in 1:m) {
d[i,8]~dbin(q[8],n[i])
}
q[8]~dbeta(1, 1)
```

```
for (i in 1:m) {
d[i,9]~dbin(q[9],n[i])
}
q[9]~dbeta(1, 1)
for (i in 1:m) {
d[i,10]~dbin(q[10],n[i])
}
q[10]~dbeta(1, 1)
for (i in 1:m) {
d[i,11]~dbin(q[11],n[i])
}
q[11]~dbeta(1, 1)
for (i in 1:m) {
d[i,12]~dbin(q[12],n[i])
}
q[12]~dbeta(1, 1)
for (i in 1:m) {
d[i,13]~dbin(q[13],n[i])
}
q[13]~dbeta(1, 1)
for (i in 1:m) {
d[i,14]~dbin(q[14],n[i])
}
q[14]~dbeta(1, 1)
for (i in 1:m) {
d[i,15]~dbin(q[15],n[i])
}
q[15]~dbeta(1, 1)
for (i in 1:m) {
d[i,16]~dbin(q[16],n[i])
}
q[16]~dbeta(1, 1)
for (i in 1:m) {
d[i,17]~dbin(q[17],n[i])
}
q[17]~dbeta(1, 1)
for (i in 1:m) {
d[i,18]~dbin(q[18],n[i])
}
q[18]~dbeta(1, 1)
for (i in 1:m) {
d[i,19]~dbin(q[19],n[i])
}
q[19]~dbeta(1, 1)
for (i in 1:m) {
d[i,20]~dbin(q[20],n[i])
}
q[20]~dbeta(1, 1)
for (i in 1:m) {
d[i,21]~dbin(q[21],n[i])
}
q[21]~dbeta(1, 1)
for (i in 1:m) {
d[i,22]~dbin(q[22],n[i])
}
```

```
q[22]~dbeta(1, 1)
for (i in 1:m) {
d[i,23]~dbin(q[23],n[i])
}
q[23]~dbeta(1, 1)
for (i in 1:m) {
d[i,24]~dbin(q[24],n[i])
}
q[24]~dbeta(1, 1)
for (i in 1:m) {
d[i,25]~dbin(q[25],n[i])
}
q[25]~dbeta(1, 1)
for (i in 1:m) {
d[i,26]~dbin(q[26],n[i])
}
q[26]~dbeta(1, 1)
for (i in 1:m) {
d[i,27]~dbin(q[27],n[i])
}
q[27]~dbeta(1, 1)
for (i in 1:m) {
d[i,28]~dbin(q[28],n[i])
}
q[28]~dbeta(1, 1)
for (i in 1:m) {
d[i,29]~dbin(q[29],n[i])
}
q[29]~dbeta(1, 1)
for (i in 1:m) {
d[i,30]~dbin(q[30],n[i])
}
q[30]~dbeta(1, 1)
for (i in 1:m) {
d[i,31]~dbin(q[31],n[i])
}
q[31]~dbeta(1, 1)
for (i in 1:m) {
d[i,32]~dbin(q[32],n[i])
}
q[32]~dbeta(1, 1)
for (i in 1:m) {
d[i,33]~dbin(q[33],n[i])
}
q[33]~dbeta(1, 1)
for (i in 1:m) {
d[i,34]~dbin(q[34],n[i])
}
q[34]~dbeta(1, 1)
for (i in 1:m) {
d[i,35]~dbin(q[35],n[i])
}
q[35]~dbeta(1, 1)
for (i in 1:m) {
d[i,36]~dbin(q[36],n[i])
```

```
}
q[36]~dbeta(1, 1)
for (i in 1:m) {
d[i,37]~dbin(q[37],n[i])
}
q[37]~dbeta(1, 1)
for (i in 1:m) {
d[i,38]~dbin(q[38],n[i])
}
q[38]~dbeta(1, 1)
for (i in 1:m) {
d[i,39]~dbin(q[39],n[i])
}
q[39]~dbeta(1, 1)
for (i in 1:m) {
d[i,40]~dbin(q[40],n[i])
}
q[40]~dbeta(1, 1)
for (i in 1:m) {
d[i,41]~dbin(q[41],n[i])
}
q[41]~dbeta(1, 1)
for (i in 1:m) {
d[i,42]~dbin(q[42],n[i])
}
q[42]~dbeta(1, 1)
for (i in 1:m) {
d[i,43]~dbin(q[43],n[i])
}
q[43]~dbeta(1, 1)
for (i in 1:m) {
d[i,44]~dbin(q[44],n[i])
}
q[44]~dbeta(1, 1)
for (i in 1:m) {
d[i,45]~dbin(q[45],n[i])
}
q[45]~dbeta(1, 1)
for (i in 1:m) {
d[i,46]~dbin(q[46],n[i])
}
q[46]~dbeta(1, 1)
for (i in 1:m) {
d[i,47]~dbin(q[47],n[i])
}
q[47]~dbeta(1, 1)
for (i in 1:m) {
d[i,48]~dbin(q[48],n[i])
}
q[48]~dbeta(1, 1)
for (j in 1: s) {
infectionrate.rank[j] <- rank(q[], j) # rank of rate j
}
}
```

list(m=227, s=48)
d[,1] d[,2] d[,3] d[,4] d[,5] d[,6] d[,7] d[,8] d[,9] d[,10]d[,11]d[,12]d[,13]d[,14]
d[,15]d[,16]d[,17]d[,18]d[,19]d[,20]d[,21]d[,22]d[,23]d[,24]d[,25]d[,26]d[,27]d[,28]
d[,29]d[,30]d[,31]d[,32]d[,33]d[,34]d[,35]d[,36]d[,37]d[,38]d[,39]d[,40]d[,41]d[,42]
d[,43]d[,44]d[,45]d[,46]d[,47]d[,48]n[]

```
0    3    3    4    8    8    0    0    9    2    0    3    4    1    19
86   0    0    8    2    1    1    12   2    13   2    3    0    2    3
37   1    0    0    9    3    0    1    5    9    2    14   4    6    0
9    0    1    287
. …………………….
0    0    0    2    31   9    0    0    15   14   0    47   31   4    28
154  0    0    10   1    0    87   10   0    64   0    31   9    0    8
76   1    0    1    5    0    0    0    12   2    1    0    2    0    0
8    0    4    603
END
```
